# Sexual dysfunction and reproductive concerns in young women with breast cancer: Type, prevalence, and predictors of problems

**DOI:** 10.1002/pon.4886

**Published:** 2018-09-27

**Authors:** Lisa Ljungman, Johan Ahlgren, Lena‐Marie Petersson, Kathryn E. Flynn, Kevin Weinfurt, Jessica R. Gorman, Lena Wettergren, Claudia Lampic

**Affiliations:** ^1^ Department of Women's and Children's Health Karolinska Institutet Solna Sweden; ^2^ Department of Oncology, Faculty of Medicine and Health Örebro University Örebro Sweden; ^3^ Regional Cancer Centre, Uppsala‐Örebro Uppsala Sweden; ^4^ Department of Neurobiology, Care Sciences and Society Karolinska Institutet Solna Sweden; ^5^ Department of Medicine Medical College of Wisconsin Milwaukee WI USA; ^6^ Department of Population Health Sciences Duke University School of Medicine Durham NC USA; ^7^ College of Public Health and Human Sciences Oregon State University Corvallis OR USA

**Keywords:** body image, breast cancer, cancer, oncology, quality of life, reproductive concerns, sexual function, young

## Abstract

**Objective:**

A dearth of studies focusing on young women (<40 years) with breast cancer have hampered the understanding of the type, prevalence, and predictors of sexual dysfunction and reproductive concerns in this population.

**Methods:**

Data were collected from 181 women (response rate = 60%) diagnosed with breast cancer approximately 2 years previously (age 21‐39) using the Swedish National Quality Registry for Breast Cancer and a survey including standardized measures of sexual dysfunction, reproductive concerns, body image, and health‐related quality of life. Multivariable logistic binary regression analyses were used to identify predictors of sexual dysfunction and reproductive concerns.

**Results:**

Sexual dysfunction in at least one domain was reported by 68% of the women, and a high level of reproductive concerns in at least one dimension was reported by 58%. Model results showed that current endocrine treatment was a significant predictor of dysfunction related to lubrication (OR 3.8, 95% CI 1.2‐12.1) and vaginal discomfort (OR 8.7, 95% CI 1.5‐51.5). Negative body image was related to satisfaction with sex life (OR 1.1, 95% CI 1.0‐1.2). A high level of reproductive concerns was predicted by a wish for (additional) children in the future (OR 3.4, 95% CI 1.1‐10.2) and by previous chemotherapy (OR 2.5, 95% CI 1.1‐5.9).

**Conclusions:**

Sexual dysfunction and reproductive concerns are common in young women with breast cancer. Current endocrine treatment, previous chemotherapy, a negative body image, and a wish for children in the future predict higher level of problems.

## 1 INTRODUCTION

Women diagnosed with breast cancer at a young age face unique challenges and demands. First, young age at diagnosis (<40 years) is linked to genetically more aggressive tumors,^1^ poorer prognosis with increased mortality, and more extensive treatments.^2^ Second, a diagnosis of breast cancer during one's reproductive years may interfere with important life goals such as building intimate relationships and having children.^3^ Also, young women may be at particular risk of sexual problems due to a higher vulnerability to hormonal changes caused by cancer treatment and more concerns about body image after, eg, breast cancer surgery.^4^, ^5^ Few studies have however investigated these important issues, resulting in a lack of knowledge regarding sexual function and reproductive concerns in young women with breast cancer.

Common breast cancer treatments such as chemotherapy and endocrine treatments may have a negative impact on fertility. Chemotherapy directly induces a risk of infertility via temporary or permanent ovarian failure and amenorrhea.^6^, ^7^ The dominating endocrine treatment used in this age group (tamoxifen) on the other hand is mainly indirectly linked to inability to conceive due to its feto‐toxicity, imposing women to avoid pregnancy during this often lengthy treatment.^8^ Reproductive concerns in the context of cancer extend beyond the biological fertility potential and include concerns about recurrence, living long enough to raise children, discussing fertility problems with a partner, acceptance of possible infertility, and concerns regarding the child's health.^9^, ^10^ This multidimensional concept was developed based on young female survivors' identification of important aspects related to reproduction and thus includes a range of concerns related to fertility and parenthood after cancer.^9^ Reproductive concerns have been associated with decreased quality of life (QoL)^3^, ^11^ and long‐term depressive symptoms in women with cancer.^12^ A wish for children, low number of prior pregnancies, prior difficulty conceiving, and being nulliparous at diagnosis have been reported to increase the risk of high levels of reproductive concerns.^10^, ^12^

Sexual dysfunction, such as a reduction in desire, lubrication difficulties, and vaginal pain and discomfort, has been reported by women with breast cancer.^4^, ^13^, ^14^, ^15^ These symptoms have in previous studies been related to a premature menopause induced by chemotherapy, which can be transient or permanent.^4^, ^15^ Results regarding the effects of endocrine treatment on sexual function have been somewhat contentious^15^ with some studies reporting no clear relationship with sexual dysfunction,^16^, ^17^, ^18^ whilst others have reported that women on endocrine treatment complain about pain, burning or discomfort during intercourse, vaginal tightness, and hot flashes.^19^, ^20^, ^21^ Recent evidence suggests ovarian suppression, which is achieved with GNRH analogues, to be particularly related to sexual dysfunction, whereas tamoxifen alone may not be related to these issues.^22^, ^23^ Importantly, few studies have examined sexual problems exclusively in young women (<40 years) with breast cancer.

Both biological factors (including hormonal alterations, pain, and fatigue) and psychological factors (such as negative body image, depression, and anxiety) have been suggested as mechanisms involved in sexual dysfunction in women with breast cancer (eg, the biopsychosocial model by Bober and Sanchez Varela, 2012). Negative body image has been reported to mediate the relationship between mastectomy and low interest in sexual activities.^4^ Similar results have been reported for young adults (<40 years) with different types of cancers where a negative perception of one's physical appearance was shown to predict sexual problems.^24^ Canada and Schover (2012) have furthermore reported that women who view themselves as infertile experience lower sexual satisfaction than women who believe they have normal fertility, pointing to the potential intricate interdependence between these issues.^25^

The overall aim of the current study was to investigate sexual dysfunction and reproductive concerns in women under the age of 40 at breast cancer diagnosis. The study also aimed to identify predictors of high levels of problems and the potential interdependence between sexual dysfunction and reproductive concerns.

## 2 METHODS

### 2.1 Participants and procedure

Participants were identified using the Swedish National Quality Registry for Breast Cancer which is a high‐quality registry with almost complete coverage.^26^ Identification criteria included women diagnosed with invasive breast cancer at the age of 18 to 39 in Sweden (diagnosis July 2014‐June 2015). Information on vital status and contact details were obtained through linkage with the Swedish population registry; women without address information were excluded. At data collection (December 2016‐January 2017), participants were 1.5 to 2.5 years post diagnosis.

Information about the study was sent by post to potential participants together with a survey and a pre‐paid envelope for questionnaire return. Two reminders were sent to non‐responders. By responding to the survey, participants consented to participate in the study. Ethical approval was obtained by the Regional Ethical Review Board in Stockholm, Sweden (Ref No: 20131746‐31/4). No compensation was given for study participation.

## 3 MEASURES

### 3.1 Clinical variables

Clinical data obtained from the registry included date of diagnosis, distant metastases, planned adjuvant cancer treatment (chemotherapy, radiation therapy, endocrine treatment, targeted therapy), and type of surgery (breast conserving vs mastectomy). Information about current cancer treatment, fertility preservation, and menstruation status was collected in the survey.

### 3.2 Socio‐demographic variables

Study specific questions were used to assess socio‐demographic information including birth country, education, employment, family situation (partner, children), and wish for (additional) children.

### 3.3 Sexual function

Sexual function was assessed using the Patient‐Reported Outcomes Measurement Information System® Sexual Function and Satisfaction measure version 2.0 (SexFS).^27^ The following five domains were selected: vaginal lubrication (two items), vaginal discomfort (four items), vulvar discomfort—clitoral (one item), vulvar discomfort—labial (1 item), and satisfaction with sex life (two items). Scores were transformed to a T‐score metric with 50 corresponding to the mean of the population of US adults who have been sexually active in the past 30 days.^27^ We considered one standard deviation (10 points on the T‐scale) under/above 50 as indicative of dysfunction. For “Satisfaction with sex life” and “Lubrication,” lower scores indicate more problems and in the three discomfort domains higher scores indicate more problems. One question concerning reasons for not having had sexual activity with a partner during the past 30 days was also included. The SexFS has shown adequate content and construct validity and test‐retest reliability.^27^, ^28^

We translated the SexFS‐v2 into Swedish and linguistically validated it in accordance with the procedure developed by FACITrans and PROMIS.^29^

### 3.4 Reproductive concerns

Reproductive concerns were assessed using the Reproductive Concerns After Cancer scale (RCAC).^9^ The RCAC is a multidimensional scale assessing a range of reproductive and parenthood concerns developed and evaluated for young adult female cancer survivors.^9^ The scale encompasses 18 items scored on a five‐point scale ranging from 1 = “Strongly disagree” to 5 = “Strongly agree”, and includes the six dimensions: “Fertility potential,” “Partner disclosure,” “Child's health,” “Personal health,” “Acceptance,” and “Becoming pregnant.” For each dimension, a mean value of >4 indicates a high level of reproductive concern.^9^ The RCAC has demonstrated satisfactory internal consistency and construct validity.^30^ The scale was independently translated into Swedish by two bilingual researchers, evaluated by two lay panels (*n* = 7) and one patient panel (*n* = 12), and in cognitive interviews with three young cancer patients.

### 3.5 Body image

Body image was assessed using the Body Image Scale (BIS) which assesses body image discomfort associated with cancer.^31^ The BIS consists of 10 items, and responses are given on a 4‐point scale, from “not at all” (0) to “very much” (3) with higher scores indicating a positive body image. The BIS has shown high test‐retest reliability and internal consistency (Cronbach's Alpha 0.93) in a sample of cancer patients.^31^ Cronbach's Alpha for the BIS in the current study was 0.91.

### 3.6 Health‐related quality of life

Health‐related QoL was measured using the EORTC QLQ‐C30 version 3.0 (QLQ‐30‐v3.0), which is a 30‐item questionnaire developed to assess the QoL of cancer patients.^32^, ^33^ The QLQ‐30 has demonstrated good psychometric properties in cancer populations.^32^, ^34^ In the current study, the summary score (higher values reflect better QoL) was used according to the EORTC QLQ‐C30 Scoring Manual (3rd Edition) (2001) and Geisinger et al (2016). Cronbach's alpha for the QLQ‐30‐v3.0 in the current study was 0.93.

### 3.7 Statistical analyses

Fisher's exact test was used to analyze differences between responders and non‐responders. Prevalence of sexual dysfunction and reproductive concerns was calculated using descriptive statistics. The relationships between the domains of the SexFS and the dimensions of the RCAC were analyzed by Pearson's correlation coefficient. Predictors for sexual dysfunction and reproductive concerns were identified using multivariable binary logistic regression with effects expressed as odds ratios (OR) using 95% confidence intervals (CI). Four models were specified with variables selected beforehand according to previous literature on potentially important factors for the outcomes. Satisfaction with sex‐life, vaginal lubrication, and vaginal discomfort were used as outcomes for sexual problems. For reproductive concerns, an overall indicator of a high level of problems (at least one dimension >4) was used. Variables included as predictors in the four models were current age (‐35y; 36‐40 y; 41‐y), education (“university degree”; “no university degree”), employment status (“full‐time”; “not‐full‐time”), birth country (“Sweden”; “other”), have children (“yes”; “no”), current partner relationship (“yes”; “no”), days since diagnosis (continuous variable), previous cancer treatment (“radiation therapy”; “chemotherapy”; “targeted therapy”), distant metastases (“yes”; “no”), mastectomy (“yes”; “no”), current endocrine treatment (“yes”; “no”), menstruation status (“regular”; “irregular”; “no menstruation past 6 months”), fertility preservation (“yes”; “no”), wish for (additional) children (“yes”; “uncertain”/“no”), satisfaction with sex life before cancer (“low”; “moderate”; “high”), BIS (continuous variable), and QLQ‐30‐summary score (continuous variable). The models were evaluated using significance level *P* < 0.05 and Nagelkerke's *R* ^2^. All the statistical analyses were performed using SPSS Statistics for Windows, version 24 (IBM Corp., Armonk, N.Y., USA).

Missing value analyses were performed by visualizing the pattern of missing data and by calculating descriptive statistics (mean, SD, frequency) per variable grouped by missing and observed values for the other variables. The missing data analyses indicated no systematic pattern, and data were thus considered unrelated to the other measured variables.

## 4 RESULTS

### 4.1 Participants

Of the 301 individuals matching the inclusion criteria, 181 returned the questionnaire representing a response rate of 60%. There were no statistically significant differences between responders and non‐responders with regard to age or previous treatment modalities. Fewer responders had distant metastases (*P* = 0.042); however, very few individuals had such metastases: two responders vs seven non‐responders.

Mean age at diagnosis was 34.6 years (SD = 4.1; range = 21‐39), and mean current age was 36.5 years (SD = 4.1; range = 23‐42). Sweden was the birth country for 81% of the participants. Mean number of days since diagnosis was 731 (SD = 103, range = 552‐915, mean equals 2 years). Satisfaction with sex life before cancer was retrospectively reported to be high by 141 (79%), moderate by 20 (11%), and low by 18 (10%) of the participants. Total score for BIS was 13.1 (SD = 7.3), and summary score for the QLQ‐30 was 76.1 (SD = 16.0). Please see Table 1 for demographics and clinical variables.

**Table 1:** Demographics and clinical variables presented by groups according to current age

n(%)	Total sample	Age	Age	Age
		≤35	36‐40	≥41
	181 (100)	51 (28)	80 (44)	50 (28)
Highest education				
University	110 (61)	26 (51)	53 (66)	31 (62)
Elementary/upper secondary/other	71 (39)	25 (49)	27 (34)	19 (38)
Employment status				
Full‐time	90 (50)	22 (43)	45 (56)	23 (46)
Other^a^	91 (50)	29 (57)	35 (44)	27 (54)
Have children	138 (77)	32 (63)	62 (80)	44 (88)
Current partner relationship	158 (87)	42 (82)	69 (86)	47 (94)
Previous cancer treatment				
Chemotherapy	117 (70)	37 (76)	52 (71)	28 (62)
Radiation therapy	133 (78)	32 (65)	61 (80)	40 (87)
Endocrine treatment	119 (71)	30 (63)	54 (72)	35 (80)
Targeted therapy	49 (29)	15 (31)	23 (30)	11 (24)
Mastectomy	99 (56)	32 (64)	43 (54)	24 (49)
Ongoing cancer treatment				
None	72 (40)	23 (46)	35 (50)	14 (28)
Chemotherapy	9 (5)	4 (8)	3 (4)	2 (4)
Radiation therapy	4 (2)	1 (2)	2 (3)	1 (2)
Endocrine treatment	91 (51)	23 (46)	35 (45)	33 (66)
Other	14 (8)	4 (8)	8 (10)	2 (4)
Current menstruations				
Regular	61 (35)	20 (39)	27 (36)	14 (29)
Irregular	36 (21)	10 (20)	16 (21)	10 (21)
No menstruation	78 (45)	21 (41)	33 (43)	24 (50)
Fertility preservation				
No	123 (68)	20 (40)	60 (75)	43 (86)
Yes eggs	37 (21)	22 (44)	13 (16)	2 (4)
Yes embryo	13 (7)	6 (12)	3 (4)	4 (8)
Wish for (additional) children				
Yes	64 (36)	34 (67)	23 (30)	7 (14)
No/uncertain	114 (64)	17 (33)	54 (70)	43 (86)

Note:

Includes part‐time employment; student; unemployment; sick‐leave; other.

### 4.2 Sexual function

Sexual dysfunction in at least one domain was reported by 68%, and dysfunction in at least two domains was reported by 38% (Table 2). The most common problem was vulvar discomfort of the labia, reported by 40%. The most common reasons why participants had not had sex with a partner during the past 30 days were “Feeling unattractive” (51%), “Too tired” (49%), and “Dryness or pain in the vagina” (36%) (Table 3).

**Table 2:** Self‐reported sexual function (SexFS) and reproductive concerns (RCAC)

*SexFS* a	Mean (SD)	Number above Cut‐Off (%)^b^^,^ c	Cronbach's Alpha
Vaginal lubrication (*n* = 149)	45.9 (9.6)	42 (28)	.85
Vaginal discomfort (*n* = 147)	52.8 (10.7)	32 (22)	.87
Vulvar discomfort clitoral (*n* = 150)	54.2 (9.6)	45 (30)	NA
Vulvar discomfort labia (*n* = 147)	54.8 (9.6)	59 (40)	NA
Satisfaction with sex life (*n* = 154)	46.8 (9.1)	49 (32)	.83
At least one domain above/below cut‐off		101 (68)	
At least two domains above/below cut‐off		58 (38)	
***RCAC** (n = 178)*			
Fertility potential	2.75 (1.32)	31 (17)	.92
Partner disclosure	2.17 (1.12)	6 (3)	.88
Child's health	3.58 (1.21)	67 (38)	.84
Personal health	3.27 (1.06)	37 (21)	.68
Acceptance	2.44 (1.09)	16 (9)	.78
Becoming pregnant	2.41 (0.90)	4 (2)	.54
At least one dimension above cut‐off		104 (58)	
At least two dimensions above cut‐off		39 (22)	

a Answered by individuals who have had sexual activity (with or without partner) during past 30 days.

b SexFS cut‐off = 1 SD above/below mean of norm population.

c RCAC cut‐off = mean > 4 per dimension.

**Table 3:** Reasons not to have sexual activity with a partner during the past 30 days

(*n* = 39)^a^	*n*(%)
Feeling unattractive	20 (51)
Too tired	19 (49)
Dryness or pain in the vagina	14 (36)
Lack of interest in sexual activity	11 (28)
Lack of partner	10 (26)
Medication affecting sexual desire	8 (21)
Concerns regarding discomfort of the clitoris or labia	6 (15)
Do not enjoy sexual activity	6 (15)
In too much pain	6 (15)
Not enough time/too busy	6 (15)
Difficulties to reach climax	5 (13)

Note.

a Question posed to participants who reported not having had sexual activity with a partner during the past 30 days. Reasons reported by less than 10%: Health concerns; Partner unavailable; Do not want to risk a pregnancy, Other.

### 4.3 Reproductive concerns

At least one dimension of high level of reproductive concerns was reported by 58%, and at least two dimensions of high levels were reported by 22% (Table 2). The most common areas of concern were “Child's health” and “Personal health,” reported by 38% and 21%, respectively.

### 4.4 Relation between sexual function and reproductive concerns

Correlations between the domains of sexual function and dimensions of reproductive concerns were small, ranging from 0.01 to −0.21. The highest correlation was observed between the SexFS domain “Satisfaction with sex‐life” and the RCAC dimension “Personal health” (Pearson *r* = −0.21).

### 4.5 Predictors of sexual dysfunction and reproductive concerns

Model results showed that endocrine treatment was a significant predictor of dysfunction related to lubrication (OR 3.8, 95% CI 1.2‐12.1) and vaginal discomfort (OR 8.7, 95% CI 1.5‐51.5). BIS was a significant predictor of dysfunction with regard to satisfaction with sex life (OR 1.1, 95% CI 1.0‐1.2), but not for the other domains of sexual problems. Predictors of reproductive concerns were a wish for more children (OR 3.4, 95% CI 1.1‐10.2) and previous chemotherapy (OR 2.5, 95% CI 1.1‐6.0). QoL was significantly related to all the domains of sexual dysfunction and reproductive concerns (Table 4).

**Table 4:** Multivariable logistic binary regression models for sexual dysfunction in three SexFS domains and for reproductive concerns in at least one dimension of the RCAC

	Vaginal Lubrication^a^		Vaginal Discomfort^b^		Satisfaction with Sex Life^c^		RCAC^d^	
Predictor variables^e^	OR	95% CI	OR	95% CI	OR	95% CI	OR	95% CI
Age								
‐35(ref)	‐	‐	‐	‐	‐	‐	‐	‐
36‐40	1.42	0.42‐4.76	0.56	0.13‐2.49	1.12	0.34‐3.72	1.25	0.43‐3.57
41‐	1.17	0.30‐4.58	**0.09** *	**0.01‐0.70**	0.55	0.13‐2.36	0.84	0.26‐2.76
Education								
‐university degree	0.95	0.35‐2.59	**8.80** *	**1.69‐45.86**	2.86	0.95‐8.60	1.59	0.67‐3.80
Wish for (additional) children	2.24	0.67‐7.48	0.48	0.08‐2.88	0.66	0.19‐2.27	**3.40** *	**1.14‐10.17**
Previous chemo‐therapy	0.58	0.22‐1.56	0.78	0.21‐2.87	1.32	0.48‐3.63	**2.51** *	**1.06‐5.95**
Current endocrine treatment	**3.80** *	**1.19‐12.10**	**8.74** *	**1.48‐51.45**	1.99	0.63‐6.25	1.26	0.55‐2.88
BIS	0.99	0.91‐1.10	0.98	0.88‐1.10	**1.09** *	**1.01‐1.18**	1.05	0.98‐1.12
QLQ‐30‐v2	**0.96** *	**0.93‐0.99**	**0.90** **	**0.85‐0.95**	**0.96** **	**0.93‐0.99**	**0.97** *	**0.94‐1.00**

Note:

Statistically significant predictors (*p* < 0.05) indicated in bold.

a Nagelkerke *R* ^2^ = .25.

b Nagelkerke *R* ^2^ = .52.

c Nagelkerke *R* ^2^ = .33.

d Nagelkerke *R* ^2^ = .28.

e Non‐significant variables: employment status, birth‐country, have children, current partner relationship, days since diagnosis, previous cancer treatment (radiation therapy, targeted therapy), distant metastases, mastectomy, menstruation status, fertility preservation, and satisfaction with sex‐life before cancer.

* *p* < 0.05.

** *p* < 0.001.

## 5 DISCUSSION

The current study shows that sexual problems and reproductive concerns are common approximately 2 years post diagnosis in young women diagnosed with breast cancer. Two in three women reported at least one domain of sexual dysfunction. This is a much higher number compared with women in the general population where 10% report having sexual problem.^35^ The prevalence of problems in this study is also higher compared with older women with breast cancer.^36^, ^37^ Our results underscore that women with breast cancer face increased risk of sexual dysfunction, and furthermore, that younger women endorse even more problems than older women with breast cancer.

Predictors of sexual dysfunction varied between the more biological/physical aspects, such as vaginal discomfort and problems with lubrication, and the psycho‐social aspects, such as satisfaction with sex life. Endocrine treatment turned out as a significant predictor of both vaginal discomfort and lubrication dysfunction, which is an important finding. Furthermore, negative body image was a significant predictor of low satisfaction with sex life. This result relates to “Feeling unattractive” being the most common reason not to have had sex with a partner. Negative body image thus seems to be a concept of relevance to sexual function in young women with breast cancer and should be considered a target for interventions aiming at improving sexual health in this population.

A high level of reproductive concerns (at least one domain) was reported by 58% of the women which is comparable to previous findings (eg, Ganz^6^). The most common areas of concern were “Child health” and “Personal health.” These figures however diverse some from previous findings reporting “Fertility potential” to be a more common concern.^30^ Our results should be seen in the context of the high degree of participants who already had children (77%), and the rather low number of participants who wished for (additional) children in the future (36%) which implies a lower level of concerns regarding ability to become pregnant. Still, the overall prevalence of reproductive concerns in our study is high pointing to the relevance of this concept also for women who already are mothers.

The predictor of reproductive concerns with the highest OR (3.5) was “a wish for (additional) children.” This is an expected finding that supports previous research in women with breast cancer^10^ and underscores the relevance of inquiring about patients' reproductive intentions in connection with oncological treatment as well as after end of initial treatment and surgery. Chemotherapy was also a significant predictor of reproductive concerns, which may reflect participants' awareness of the negative impact chemotherapy has on fertility.^38^

There was a weak relationship between sexual dysfunction and reproductive concerns, and few shared predictors, suggesting separate mechanisms to be involved in these issues. Reproductive concerns may be more related to psychological variables such as proneness to worry or level of tolerance of uncertainty, whereas sexual dysfunction may be predominantly of physical origin. The identification of BIS as a predictor of dysfunction related to satisfaction with sex life points to involvement of psychological mechanisms in *some* aspects of sexual function. These mechanisms should be evaluated further in future studies.

### 5.1 Study limitations

This study has important methodological strengths as well as weaknesses. First, the use of the Swedish National Quality Registry for Breast Cancer is a strength as it allowed collection of high quality data for a year cohort of women with breast cancer in Sweden. The response rate was also acceptable compared with similar studies, and analyses of non‐responders revealed no correlation with background variables. Still, the risk of selection bias should be considered when interpreting the results. A methodological weakness is that the cut‐off for sexual dysfunction was chosen in the absence of evidence‐based consensus for this threshold and, furthermore, that the American norms might not correspond exactly to the Swedish population. Even though aspects such as age for sexual debut are similar between the countries,^39^ reliable norm data should be collected to improve quality of comparisons. Also, we used self‐reported information about current endocrine treatment to obtain valid data on actual intake, however did not specify type of treatment. This is a limitation hampering conclusions regarding effects on sexual functions by the different types of endocrine treatments. Lastly, even though the clinical data was collected before study participation, information about sexual function, reproductive concerns, body image, and QoL were collected simultaneously which hinders inferences about causality. Future longitudinal studies are encouraged to investigate the causal relationships also between QoL, body image, sexual function, and reproductive concerns.

### 5.2 Clinical implications

The current study shows that sexual dysfunction and reproductive concerns are common issues among women under the age of 40 at breast cancer diagnosis. These results support the notion that young women with breast cancer face specific challenges from both a medical and a psychosocial perspective, which should be acknowledged in clinical practice. The results also show that endocrine treatment predicts sexual dysfunction, which implies the need for adequate time for consultation in relation to this treatment, including discussion of aids that can be used to alleviate potential sexual problems. Furthermore, study results indicate that reproductive concerns should be inquired in particular following chemotherapy and among women who have a wish for children in the future. Women suffering from high levels of concerns should be offered psychosocial interventions accordingly.

## References

[1] Anders CK , Hsu DS , Broadwater G , et al. Young age at diagnosis correlates with worse prognosis and defines a subset of breast cancers with shared patterns of gene expression. J Clin Oncol. 2008;26(20):3324‐3330. 18612148 10.1200/JCO.2007.14.2471

[2] Fredholm H , Eaker S , Frisell J , Holmberg L , Fredriksson I , Lindman H . Breast cancer in young women: poor survival despite intensive treatment. PLoS One. 2009;4(11):1‐9. 10.1371/journal.pone.0007695 PMC2770847 19907646

[3] Duffy C , Allen S . Medical and psychosocial aspects of fertility after cancer. Cancer J [Internet]. 2009;15(1):27‐33. 19197170 10.1097/PPO.0b013e3181976602 PMC2719717

[4] Burwell SR , Case LD , Kaelin C , Avis NE . Sexual problems in younger women after breast cancer surgery. J Clin Oncol. 2006;24(18):2815‐2821. 16782919 10.1200/JCO.2005.04.2499

[5] Stead ML . Sexual dysfunction after treatment for gynaecologic and breast malignancies. Curr Opin Obstet Gynecol. 2003;15(1):57‐61. 12544503 10.1097/00001703-200302000-00009

[6] Ganz PA , Greendale GA , Petersen L , Kahn B , Bower JE . Breast cancer in younger women: reproductive and late health effects of treatment. J Clin Oncol. 2003;21(22):4184‐4193. 14615446 10.1200/JCO.2003.04.196

[7] Vassilakopoulou M , Boostandoost E , Papaxoinis G , de La Motte Rouge T , Khayat D , Psyrri A . Anticancer treatment and fertility: effect of therapeutic modalities on reproductive system and functions. Crit Rev Oncol Hematol [Internet]. 2015;97:328‐334. 26481950 10.1016/j.critrevonc.2015.08.002

[8] Benedict C , Thom B , Teplinsky E , Carleton J , Kelvin JF . Family‐building after breast cancer: considering the effect on adherence to adjuvant endocrine therapy. Clin Breast Cancer [Internet]. 2017;17(3):165‐170. 28087390 10.1016/j.clbc.2016.12.002

[9] Gorman JR , Su HI , Pierce JP , Roberts SC , Dominick SA , Malcarne VL . A multidimensional scale to measure the reproductive concerns of young adult female cancer survivors. J Cancer Surviv [Internet]. 2014;8(2):218‐228. 24352870 10.1007/s11764-013-0333-3 PMC4016119

[10] Partridge AH , Gelber S , Peppercorn J , et al. Web‐based survey of fertility issues in young women with breast cancer. J Clin Oncol. 2004;22(20):4174‐4183. 15483028 10.1200/JCO.2004.01.159

[11] Benedict C , Thom B , Friedman DN , Pottenger E , Raghunathan N , Kelvin JF . Fertility information needs and concerns post‐treatment contribute to lowered quality of life among young adult female cancer survivors. Support Care Cancer [Internet]. 2018;26(7):2209‐2215. 29387996 10.1007/s00520-017-4006-z PMC5984121

[12] Gorman JR , Malcarne VL , Roesch SC , Madlensky L , Pierce P . Depressive symptoms among young breast cancer survivors: the importance of reproductive concerns. Breast Cancer Res Treat. 2010;123(2):477‐485. 20130979 10.1007/s10549-010-0768-4 PMC2888956

[13] Bober SL , Sanchez Varela V . Sexuality in adult cancer survivors: challenges and intervention. J Clin Oncol. 2012;30(30):3712‐3719. 23008322 10.1200/JCO.2012.41.7915

[14] Howard‐Anderson J , Ganz PA , Bower JE , Stanton AL . Quality of life, fertility concerns, and behavioral health outcomes in younger breast cancer survivors: a systematic review. J Natl Cancer Inst. 2012;104(5):386‐405. 22271773 10.1093/jnci/djr541

[15] Gilbert E , Ussher JM , Perz J . Sexuality after breast cancer: a review. Maturitas [Internet]. 2010;66(4):397‐407. 20439140 10.1016/j.maturitas.2010.03.027

[16] Rosenberg SM , Tamimi RM , Gelber S , et al. Treatment‐related amenorrhea and sexual functioning in young breast cancer survivors. Cancer. 2014;120(15):2264‐2271. 24891236 10.1002/cncr.28738 PMC4116329

[17] Mortimer BJE , Boucher L , Baty J , Knapp DL , Ryan E , Rowland JH . Effect of tamoxifen on sexual functioning in patients with breast cancer. J Clin Oncol. 1999;17(5):1488‐1492. 10334535 10.1200/JCO.1999.17.5.1488

[18] Ganz PA . Impact of tamoxifen adjuvant therapy on symptoms, functioning, and quality of life. JNCI Monogr [Internet]. 2001;30:130‐134. 10.1093/oxfordjournals.jncimonographs.a003450 11773306

[19] Ganz BPA , Desmond KA , Belin TR , Meyerowitz BE , Rowland JH . Predictors of sexual health in women after a breast cancer diagnosis. J Clin Oncol. 1999;17(8):2371‐2380. 10561299 10.1200/JCO.1999.17.8.2371

[20] Berglund G , Nystedt M , Bolund C , Sjoden PO , Rutquist LE . Effect of endocrine treatment on sexuality in premenopausal breast cancer patients: a prospective randomized study. J Clin Oncol [Internet]. 2001;19(11):2788‐2796. 11387349 10.1200/JCO.2001.19.11.2788

[21] Day R . Quality of life and tamoxifen in a breast cancer prevention trial: a summary of findings from the NSABP P‐1 study. Ann N Y Acad Sci [Internet]. 2001;949:143‐150. 11795346

[22] Saha P , Regan MM , Pagani O , et al. Treatment efficacy, adherence, and quality of life among women younger than 35 years in the international breast cancer study group TEXT and SOFT adjuvant endocrine therapy trials. J Clin Oncol. 2017;35(27):3113‐3122. 28654365 10.1200/JCO.2016.72.0946 PMC5597253

[23] Ribi K , Luo W , Bernhard J , et al. Adjuvant tamoxifen plus ovarian function suppression versus tamoxifen alone in premenopausal women with early breast cancer: patient‐reported outcomes in the suppression of ovarian function trial. J Clin Oncol. 2016;34(14):1601‐1610. 27022111 10.1200/JCO.2015.64.8675 PMC4872319

[24] Wettergren L , Kent EE , Mitchell SA , et al. Cancer negatively impacts on sexual function in adolescents and young adults: the AYA HOPE study. Psychooncology. 2017;26(10):1632‐1639. 27240019 10.1002/pon.4181 PMC7239373

[25] Canada A , Schover L . The psychosocial impact of interrupted childbearing in long‐term female cancer survivors. Psychooncology. 2012;21(2):134‐143. 22271533 10.1002/pon.1875 PMC3123665

[26] Emilsson L , Lindahl B , Köster M , Lambe M , Ludvigsson JF . Review of 103 Swedish healthcare quality registries. J Intern Med. 2015;277(1):94‐136. 25174800 10.1111/joim.12303

[27] Weinfurt KP , Lin L , Bruner DW , et al. Development and initial validation of the PROMIS® sexual function and satisfaction measures version 2.0. J Sex Med [Internet]. 2015;12(9):1961‐1974. 26346418 10.1111/jsm.12966

[28] Flynn KE , Jeffery DD , Keefe FJ , et al. Sexual functioning along the cancer continuum: focus group results from the patient‐reported outcomes measurement information system (PROMIS™). Psychooncology. 2011;20(4):378‐386. 20878833 10.1002/pon.1738 PMC3013236

[29] Strandquist J , Eriksson L , Winterling J , et al. Translation of selected items of the PROMIS SexFS v2.0 into Swedish. In: The Second PHO Conference, October 23‐24. Copenhagen; 2016.

[30] Gorman JR , Su HI , Roberts SC , Dominick SA , Malcarne VL . Experiencing reproductive concerns as a female cancer survivor is associated with depression. Cancer. 2015;121(6):935‐942. 25377593 10.1002/cncr.29133 PMC4352116

[31] Hopwood P , Fletcher I , Lee A , Al Ghazal S . A body image scale for use with cancer patients. Eur J Cancer [Internet]. 2001;37(2):189‐197. 11166145 10.1016/s0959-8049(00)00353-1

[32] Aaronson NK , Ahmedzai S , Bergman B , et al. The European organisation for research and treatment of cancer QLQ‐C30: a quality‐of‐life instrument for use in international clinical trials in oncology. J Natl Cancer Inst. 1993;85(5):365‐376. 8433390 10.1093/jnci/85.5.365

[33] Giesinger JM , Kieffer JM , Fayers PM , et al. Replication and validation of higher order models demonstrated that a summary score for the EORTC QLQ‐C30 is robust. J Clin Epidemiol [Internet]. 2016;69:79‐88. 26327487 10.1016/j.jclinepi.2015.08.007

[34] Shih CL , Chen CH , Sheu CF , Lang HC , Hsieh CL . Validating and improving the reliability of the EORTC QLQ‐C30 using a multidimensional Rasch model. Value Health [Internet]. 2013;16(5):848‐854. 23947980 10.1016/j.jval.2013.05.004

[35] Flynn KE , Lindau ST , Lin L , et al. Development and validation of a single‐item screener for self‐reporting sexual problems in U.S. adults. J Gen Intern Med. 2015;30(10):1468‐1475. 25893421 10.1007/s11606-015-3333-3 PMC4579234

[36] Fobair P , Stewart SL , Chang SB , D'Onofrio C , Banks PJ , Bloom JR . Body image and sexual problems in young women with breast cancer. Psychooncology [Internet]. 2006;15(7):579‐594. 16287197 10.1002/pon.991

[37] Raggio GA , Butryn ML , Arigo D , Mikorski R , Palmer SC . Prevalence and correlates of sexual morbidity in long‐term breast cancer survivors. Psychol Health [Internet]. 2014;29(6):632‐650. 24404999 10.1080/08870446.2013.879136 PMC5521019

[38] Meirow D , Nugent D . The effects of radiotherapy and chemotherapy on female reproduction. Hum Reprod Update. 2001;7(6):535‐543. 11727861 10.1093/humupd/7.6.535

[39] Danielsson M , Berglund T , Forsberg M , Larsson M , Rogala C , Tydén T . Health in Sweden: the national public health report 2012. Chapter 9. Scand J Public Health. 2012;40(Suppl 9):176‐196. 23238407 10.1177/1403494812459600

